# Reporting Iraqi civilian fatalities in a time of war

**DOI:** 10.1186/1752-1505-3-9

**Published:** 2009-11-06

**Authors:** Schuyler W Henderson, William E Olander, Les Roberts

**Affiliations:** 1 Department of Psychiatry, Columbia University, New York, New York, USA; 2 Mailman School of Public Health, Columbia University, New York, New York, USA; 3 Department of Population and Family Health, Columbia University, New York, New York, USA

## Abstract

**Background:**

In February, 2007, the Associated Press (AP) conducted a poll of 1,002 adults in the United States about their attitudes towards the war in Iraq. Respondents were remarkably accurate estimating the current death toll of US soldiers, yet were grossly inaccurate in estimating the current death toll of Iraqi civilians. We conducted a search of newspapers reports to determine the extent of the discrepancy between reporting Coalition and Iraqi civilian deaths, hypothesizing that there would be an over-representation of Coalition deaths compared to Iraqi civilian deaths.

**Methods:**

We examined 11 U.S. newspapers and 5 non-U.S. newspapers using electronic databases or newspaper web-archives, to record any reports between March 2003 and March 2008 of Coalition and Iraqi deaths that included a numeric indicator. Reports were described as "events" where they described a specific occurrence involving fatalities and "tallies" when they mentioned the number of deaths over a period of time. We recorded the number of events and tallies related to Coalition deaths, Iraqi civilian deaths, and Iraqi combatant deaths

**Results:**

U.S. newspapers report more events and tallies related to Coalition deaths than Iraqi civilian deaths, although there are substantially different proportions amongst the different U.S. newspapers. In four of the five non-US newspapers, the pattern was reversed.

**Conclusion:**

This difference in reporting trends may partly explain the discrepancy in how well people are informed about U.S. and Iraqi civilian fatalities in Iraq. Furthermore, this calls into question the role of the media in reporting and sustaining armed conflict, and the extent to which newspaper and other media reports can be used as data to assess fatalities or trends in the time of war.

## Background

In February, 2007, the Associated Press (AP) conducted a poll of 1,002 adults in the United States about their attitudes towards the war in Iraq [1]. Respondents were asked to estimate the number of US soldiers and Iraqis who had died in the war. The median estimate for US soldiers was 2,974 while the actual toll at the time was 3,100 [2]. In contrast, when asked to estimate the number of Iraqi civilian casualties, the median answer was 9,890 at a time when it was at least 10 times this number, with some estimates putting the toll at 50 times this number [3,4]. The U.N. Assistance Mission for Iraq, for example, tallied more than 34,000 deaths in 2006 alone [5].

This poll suggests that Americans were remarkably accurate in estimating the numbers of US military who had died but were inaccurate in estimating the numbers of Iraqis who had died. We conducted a search of newspapers to determine the extent of the discrepancy between reporting Coalition and Iraqi civilian deaths (see appendix 1 for examples), hypothesizing that there would be an over-representation of Coalition deaths compared to Iraqi civilian deaths, reflecting the discrepancy in broader public opinion. This paper reports the relative numbers of articles describing Coalition deaths and Iraqi deaths for 11 US papers, and five non-US newspapers.

## Methods

Newspapers were selected by having each participant review their hometown newspaper. Five non-US newspapers were also selected: one (*The Guardian*) was selected as a hometown paper. A second contributor picked a middle-eastern paper because his home town (NYC) was already taken. The results were so dramatically different that others were sought: they constituted all of the Middle East newspapers we could find that were free, online, and had a full text search mechanism such as ProQuest. By reviewing the group's hometown newspaper, we examined a geographically diverse selection of US newspapers: *San Francisco Chronicle, New York Daily News, Columbus Dispatch, Christian Science Monitor, Pittsburgh Post-Gazette, Los Angeles Times, Dayton Daily, San Antonio Express, Boston Globe, Bergen Record*, and *Washington Times *(n = 11), as well as five non-US newspapers: the *Lebanon Daily Star, Gulf News, Turkish Daily News, Al-Bawaba *and *The Guardian *(n = 5).

For each month between the start of the second Gulf War (March, 2003) and March, 2008, using the search language IRAQ and KILL* or IRAQ and DEATH* in either ProQuest or Factiva databases, we recorded how often each newspaper reported death events and/or overall death tallies in the body of text. For a couple of newspapers with their own web archives, we used the same search methodology. This search procedure would result in a list of articles with either Iraq and any word beginning with "kill" or Iraq and any word beginning with "death", or both. We defined an "event" as any description of a violent death and defined a "tally" as any description of a death toll in a time period. We further classified these events or tallies as "Coalition", "Iraqi Civilian", "Combatant" or "Other" (for vague/ambiguous descriptions). Many articles included both events and tallies and thus could have been recorded in multiple categories. For example, a report that mentioned the death of a coalition soldier and added that this raised the death toll for coalition soldiers that week to ten would count as both an event and a tally. While these terms may not catch every article of interest, it was felt that using consistent terms would still provide an accurate measure of the relative numbers of Coalition vs. Iraqi death events and tallies.

Participants were asked to record the number of four kinds of articles that occurred in their hometown paper between March 2003 and March of 2008: those that described at least one event where a Coalition member was killed, those that described at least one event where an Iraqi was killed (further divided into Combatant and Civilian), articles that provided a tally of Coalition deaths for some time period, and articles that provided a tally of Iraqi deaths for some time period. While we agreed on broad definitions such as all police and non-military government employees would be called civilians, difficult judgment calls (e.g. military recruits that were killed but were not yet in the employ of the military) were left up to the judgment of the reviewer. It was felt that by having people raised in the various towns of the newspapers, reading and judging the reports with a local perspective, we would get the closest reflection of the experience of those papers' readers.

If an event was reported more than once in the same newspaper, we counted it every time it was reported, so long as it was in a separate article. If a single soldier's death was reported numerous times, we counted each time individually (for example, if a soldier's death was mentioned in a news report, an obituary, and an editorial, it was counted three times).

For death events, the sum of the articles seen in each month was tallied among the US and Middle-Eastern newspapers. While this may have the effect of over-influencing the results by the papers with the most death event articles (e.g. the *Los Angeles Times*), these papers tended to have the largest readership and thus we believe the summation is somewhat self-correcting in terms of nationwide reader experience.

## Results

### Events

**Figure 1:**
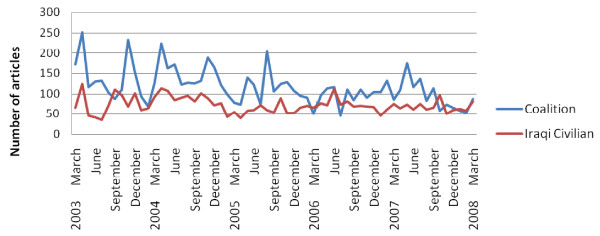
**Number of articles per month reporting Coalition and Iraqi civilian deaths in 11 US newspapers**.

**Figure 2:**
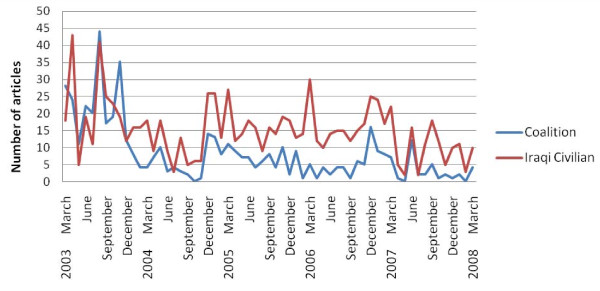
**Number of articles per month reporting Coalition and Iraqi civilian deaths in four Middle-east newspapers**.

The five year review of 11 US newspapers produced 7,151 articles describing Coalition deaths, 4,445 articles describing Iraqi Civilian deaths. The 4 Middle-Eastern Newspapers produced 495 articles describing Coalition deaths, and 923 articles describing Iraqi Civilian deaths. The summation of the number of Coalition and Iraqi Civilian death events is presented in the figures below, first for 11 US newspapers, and second for 4 Middle-Eastern Newspapers (Figure 1 and Figure 2).

### Tallies

**Figure 3:**
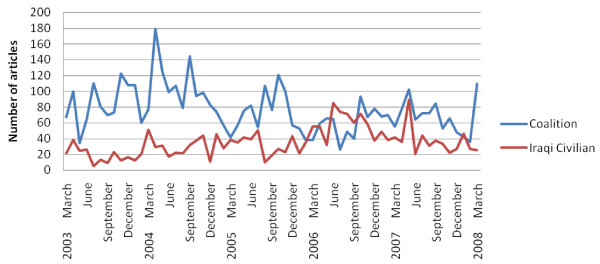
**Number of articles per month reporting Coalition and Iraqi civilian death tallies in 11 US newspapers**.

**Figure 4:**
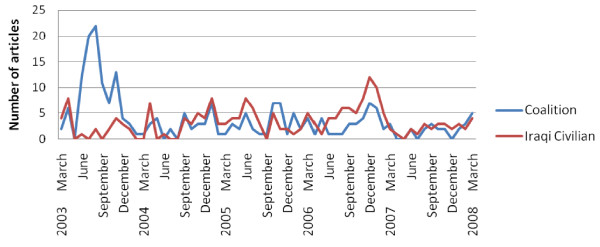
**Number of articles per month reporting Coalition and Iraqi civilian death tallies in four Middle-east newspapers**.

The five year review of 11 US newspapers produced 4,701 articles describing Coalition death tallies and 1,972 articles describing Iraqi Civilian death tallies. The 4 Middle-Eastern newspapers produced 109 articles describing Coalition death tallies and 112 articles describing Iraqi Civilian death tallies. The summation of the number of Coalition and Iraqi Civilian death tallies is presented in the figures below, first for 11 US newspapers, and second for 4 Middle-Eastern Newspapers (Figure 3 and Figure 4).

### Ratios

**Figure 5:**
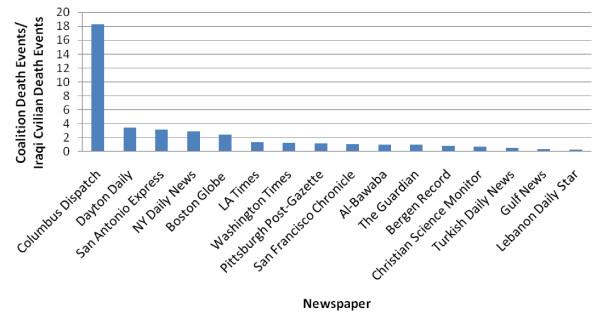
**Ratio of articles reporting Coalition deaths to Iraqi civilian deaths in examined papers, March 2003 - March 2008**.

**Figure 6:**
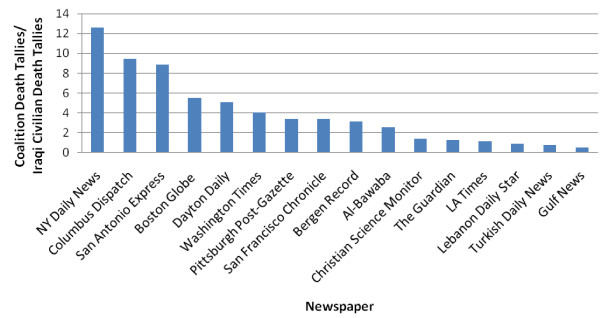
**Ratio of articles reporting Coalition death tallies to Iraqi civilian death tallies in examined papers, March 2003 - March 2008**.

Figures 5 and 6 show the ratios of Coalition to Iraqi civilian death articles in the studied newspapers (Figure 5 and Figure 6).

## Discussion

How the media chooses to report the numbers of dead and dying in Iraq may play a role in shaping the public's knowledge about the consequences of the war. The AP poll showed that respondents were substantially more accurate in identifying the number of Americans who had died as a result of the Iraq war than Iraqis. There are several striking findings in our study. First, by even the most conservative estimates, at least 20 times as many Iraqis have died as Coalition members, but in virtually all American papers there are far more articles describing coalition death events and tallies. Second, this relationship was reversed for three of the four Middle-Eastern papers and the one British paper included. Third, there are substantial differences between U.S. newspapers.

To the best of our knowledge, there is no other study that has quantified how the media has represented deaths in the time of war, or compared US newspapers to newspapers in the Middle East. The discrepancies we have observed should encourage further study into how media representation affects public knowledge, attitudes towards wars, and public debates about the consequences of a war. Our study cannot and does not explain how or why there is a variance in reporting and nor does it measure accuracy *per se*. Certainly all newspapers can only report some fraction of killings in a country as vast and violent as Iraq. But specific factors seem to be influencing the level and the relative reporting of victims from the different nationalities involved. These factors may include: local interests; local sensitivities, such as residents who have lost relatives; wire service affiliations; access to embedded journalist reports; the economic and political agendas of the various editorial boards, and other political pressures.

This study poses a challenge to journalism. Mark Twain once said to a class of Army cadets, "By the etiquette of war, it is permitted to no one below the rank of newspaper correspondent to dictate to the general in the field." [6] While his joke suggests that journalists tend to assume an expertise they may not deserve, there is an important point contained therein: in a democracy, the military operates on behalf of the people, who in turn are in a large part informed by their media outlets. Journalists risk their lives to report on wars and face numerous ethical challenges - from the ethics of what to report to the practicalities of being embedded. However, despite what individual journalists may achieve, there can be an overall effect from the media as a whole, and our study appears to show that Americans will likely be exposed to one story (of American fatalities) with much less information about another story (of Iraqi fatalities). Given the discrepancy between the U.S. and the non-U.S. newspapers, it would appear that political and cultural factors may be playing a role in how these stories are being told.

This study has a number of significant limitations. We cannot assume that media reports of death events/tallies correlates with overall knowledge, especially if people obtain their news from numerous sources. Respondents do not only get their news from newspapers. At about the same time as the AP poll was conducted, Harris Interactive conducted a poll in which they asked about how Americans get their news [7]. The vast majority (77%) said they watch local broadcast news and 71% said they watched network broadcasts or cable news several times a week or daily. 63% reported reading a local daily newspaper and 18% read a national newspaper several times a week or daily. 64% go online to get news (which may be from newspapers online). Thus, a limitation of our study is a focus on newspapers, rather than broadcast news, and our exclusion of national newspapers such as *Herald Tribune *or *USA Today*. A review of TV and radio reports would be far less easily quantified because length of segments varies widely and visual images of killed or injured may be far more memorable or exert more influence.

In terms of study design, this study involved a number of participants each tallying scores from a newspaper; this required individual judgment as to what constituted an "event" and a "tally"; discussions amongst the participants, usually over possibly ambiguous reports, suggested that although this remains a limitation, the reliability and validity of these terms was high with little variation between reviewers. Furthermore, while it is not typical of study designs to have a flexible set of "definitions", we feel this review method captures the at-home readers' experience more robustly and best interprets the narrative component of war reporting.

While we believe our search methods capture a majority of the articles written about deaths in the Iraqi war, our search language did not include every mention of the war. It excludes non-specific, general remarks such as "the civilian death toll is rising". We explored many different search terms initially and these two pairs gave us a comparatively high number of hits with a low fraction of articles with no events or tallies. Of note, we were not expecting to capture every article, just the vast majority with a consistent process that could detect trends over time and between papers. Finally, it is worth remembering that other means of communicating in the newspaper, including editorials and photographs, may be discussing or evoking the war compellingly but without the numeric indicators that would place them within our study.

## Conclusion

The initial AP poll reported that a substantial number of the respondents - 17% - knew someone who had been injured or killed in the war and 65% knew someone personally who was currently or had previously served in the war [2]. It is expected that many Americans have a personal investment in the war and so may have been more attuned to statistics about the war pertaining to U.S. military. As papers reflect the interests of their readers, it is not surprising that U.S. newspapers describe more events and tallies related to Coalition deaths than Iraqi civilians. Nevertheless, it is somewhat inconsistent with the goals of journalism that among violent deaths in Iraq, it appears more likely an event will be reported in U.S. newspapers if the decedent is an American. We feel that this study casts an important light on the role of the media in representing war, and may explain in part why Americans are so well informed about Coalition deaths and relatively ignorant about Iraqi deaths. This paper calls into question the extent to which the media plays a role in promoting and sustaining armed conflict, and even whether or how the media can be a tool for civilian check on military action or complicit in ongoing warfare [8].

Finally, this work poses a significant challenge to those databases, including the Iraq Body Count http://www.iraqbodycount.org. and the Uppsala Conflict Data Set http://www.pcr.uu.se/research/UCDP/index.htm, that assess conditions and fatalities based on media reports. Newspaper data is not impartial or proven and our study strongly suggests that there are contextual, including possibly cultural and political, factors that profoundly affect what and how data regarding fatalities in the Iraq War are being presented.

## Competing interests

The authors declare that they have no competing interests.

## Authors' contributions

LFR proposed the review and supervised data collection. SWH wrote much of the initial results and collected data. WEO prepared the figures and collected data. All authors read and approved the manuscript.

## Appendix 1

The following are two examples of our search rules:

In an article from the New York Daily News on July 7, 2007: "*A suicide bomber killed more than 100 people by detonating a truck bomb in the center of an Iraq market yesterday, police said. The killings pushed the death toll in the last three days to more than 140 people - including eight U.S. soldiers. Local police said the bombing close to Tuz Khurmatu, north of Baghdad, killed 105 and left more than 250 wounded ..." *Under our search rules, we would record this article as one "Coalition" death incident article and one "Iraqi Civilian" incident article. Because this tally did not cover a specific month, week or year, it was not recorded as a tally.

In an article from the New York Daily News on May 19, 2007: "*After a week with the anguish of not knowing whether their sons were alive or dead, four families of troops from New York's 10th Mountain Division learned yesterday which of them can still hold on to hope ... in Iraq yesterday, five more U.S. troops were killed, three by a roadside bomb northeast of Baghdad. Two others were killed and nine wounded in separate attacks in southern sectors of the capital. The latest casualties brought the U.S. death toll for May to at least 58, and to 3,408 since March 2003..." *Under our search rules, we would record this article as one "Coalition" death incident article and one "Coalition" death tally article.
